# Evaluation of an Online Survey for Pertussis Case Investigations in Regional Queensland: Impacts on Workload and Disease Trends

**DOI:** 10.3390/tropicalmed10090260

**Published:** 2025-09-12

**Authors:** Ho Yeung Lam, Arifuzzaman Khan, Matthew O’Bryan, Michelle Jones, Josette Chor

**Affiliations:** Wide Bay Public Health Unit, Wide Bay Hospital and Health Service, Queensland Health, Hervey Bay, QLD 4655, Australia

**Keywords:** pertussis, whooping cough, disease surveillance, public health, online survey, digital health, interrupted time series analysis

## Abstract

In 2024, a significant pertussis surge in Queensland, Australia, strained public health resources. To improve investigation efficiency, the Wide Bay Public Health Unit introduced an online survey for pertussis cases on 1 August 2024, which collected data and provided automated health advice. This retrospective evaluation assessed survey acceptance and staff workload. A controlled interrupted time series (ITS) analysis compared pertussis incidence in the intervention region against a control group of four other de-identified regional hospital and health services in Queensland where the survey was not implemented. Of the 298 eligible cases, 140 responded (47.0%); a total of 67.9% of these required no further staff follow-up, a subgroup for whom time savings were statistically significant (*p* < 0.01). The ITS analysis for the total population revealed a significant 4.9% weekly reduction in the notification trend compared to the control group (Rate Ratio: 0.95, *p* = 0.001), with a non-significant immediate level change. The online survey is a practical and effective tool for pertussis investigation in a regional setting. It is associated with significant workload efficiencies and a favourable, statistically significant impact on community disease transmission trends.

## 1. Introduction

Pertussis, commonly known as whooping cough, is a highly contagious respiratory infection caused by *Bordetella pertussis*. Despite being vaccine-preventable, pertussis has re-emerged in recent decades across different countries, largely due to waning immunity and suboptimal vaccine coverage [1]. This re-emergence, coupled with its characteristic pattern of epidemic cycles every 3 to 5 years, highlights the need for vigilant surveillance and prompt public health interventions to limit its transmission [2].

**Figure 1:**
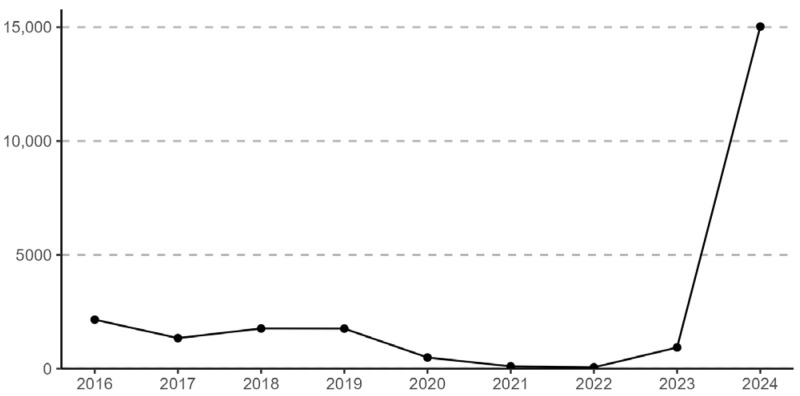
Annual number of pertussis notifications in Queensland, 2016–2024 [4].

In Queensland, Australia, pertussis is a notifiable condition under the Public Health Act 2005. Public Health Units (PHUs) are responsible for the investigation and management of notified cases through phone interviews, risk assessments, and implementation of control measures to prevent further transmission [3]. In 2024, similar to the rest of Australia, Queensland experienced an unprecedented surge in pertussis notifications, with over 15,000 cases, far exceeding the preceding few years during the COVID-19 pandemic as well as the pre-pandemic levels (Figure 1) [4]. This dramatic rise placed considerable strain on PHUs across the state, particularly smaller units in regional and rural areas, which often operate with limited resources while also managing a broad range of other public health priorities.

In recent years, there has been growing interest in leveraging digital technology to support communicable disease control efforts [5], one such innovation being the use of online surveys. Potentially, online surveys can streamline case investigations by automating some of the routine tasks, reducing the administrative burden on public health staff, and improving the effectiveness and timeliness of risk communication and public health response [6].

In response to the surge in pertussis notifications, the Wide Bay Public Health Unit (WBPHU), which serves a population of over 234,000 across an area of approximately 370,000 km^2^ in regional Queensland, implemented an online survey tool in August 2024 to support pertussis case investigation and management. This evaluation aimed to assess the integration and utility of the online survey, inform future improvements to the tool, and contribute to future utilisation of digital innovations in communicable disease control, particularly in regional settings.

## 2. Materials and Methods

### 2.1. Online Survey Design and Implementation

The online survey was launched on 1 August 2024 using the Queensland Health Microsoft Forms platform (Microsoft Corporation, Redmond, WA, USA). It was designed by WBPHU based on the existing pertussis case investigation form [3]. The 10 min survey collected information on personal details, symptoms, clinical and vaccination history, and exposure and contact history, as well as antibiotic treatment. Health advice regarding pertussis treatment and prevention of further transmission was provided during the survey.

All pertussis cases notified to WBPHU through the statewide Notifiable Conditions System (NoCS) were first assessed for eligibility based on the availability of a valid mobile phone number and the age of the case. The age eligibility criteria were progressively expanded during the rollout period: from 1 August 2024, cases aged over 16 years were eligible; from 5 August, eligibility was lowered to those over 12 years. From 22 August, all cases aged over five years became eligible, and from 13 December, there were no age restrictions. Eligible cases received a Short Message Service (SMS) invitation with a hyperlink to the survey within one business day of notification. Survey questions were customised depending on whether the respondent was the patient or a parent or guardian responding on behalf of their child. To streamline the investigation process, automated prompts were added to the survey on 5 September. Respondents who had not been prescribed antibiotics or had received inappropriate antibiotics treatment were prompted to seek medical advice from their healthcare provider. Those who were on appropriate antibiotics were reminded to complete the full course.

All survey responses were reviewed by WBPHU staff and matched to the corresponding NoCS notification using the personal information provided. Red flags were defined as high-risk exposures during the infectious period, including household contacts with women in the last month of pregnancy or children under six months of age, or attendance at high-risk settings such as childcare centres, preschools, schools, residential care facilities, or healthcare facilities (excluding attendance for the current pertussis illness). If red flags were detected, staff would contact the case by phone for additional information and follow up actions. No further follow-up would be conducted in the absence of red flags. If no survey response was received within two business days, the case would be contacted for routine phone interviews (Figure S5).

### 2.2. Evaluation Aims and Objectives

This retrospective service evaluation aimed to assess the integration of an online survey tool into the pertussis case investigation process and identify opportunities for improvement. The objectives of the evaluation are summarised in Table 1.

### 2.3. Data Collection and Analysis

Data on individual pertussis cases were obtained from three sources: (1) notifications recorded in the statewide NoCS between 1 January 2024 and 31 December 2024; (2) WBPHU case follow-up records; and (3) online survey response data (between 1 August 2024 and 31 December 2024). Additionally, we conducted a short survey with other Queensland PHUs to gather information on their approaches to managing pertussis notifications during 2024. Data was analysed in accordance with the study objectives (Table 1).

All personally identifiable information was anonymised prior to analysis. Analyses were conducted using R (version 4.4.2) [7]. Data manipulation and visualisation were performed using the tidyverse suite of packages (version 2.0.0) [8]. The segmented quasi-Poisson regression models were fitted using functions from the stats (version 4.4.2) and MASS (version: 7.3-65) packages [9], and final tables were generated using the flextable package (version: 0.9.7) [10]. Descriptive statistics, including proportions, medians, and interquartile ranges, were used to summarise relevant metrics, and the Mann–Whitney U test was applied for inferential comparisons (Table 1). To evaluate the impact of the online survey on pertussis incidence, controlled interrupted time series (ITS) analyses were conducted. These analyses compared the weekly incidence rates of pertussis notification per 100,000 population in the intervention group, Wide Bay Hospital and Health Service (WBHHS), with a control group comprising a combination of four other de-identified regional Hospital and Health Services (HHSs) where no such online survey was implemented. Analyses were performed for both the overall population and for children over five years of age, reflecting the primary target group for survey invitations. The period from 1 to 22 August 2024 was excluded from the ITS analysis as a buffer period to allow for full survey implementation. A segmented Poisson regression model was utilised, with adjustments made to account for potential autocorrelation and seasonality in the data.

### 2.4. Ethical Approval

This study was granted an exemption from human research ethics review by the Coronial and Public Health Sciences Human Ethics Committee, Queensland Health (EC00305).

## 3. Results

### 3.1. Survey Responses

**Figure 2:**
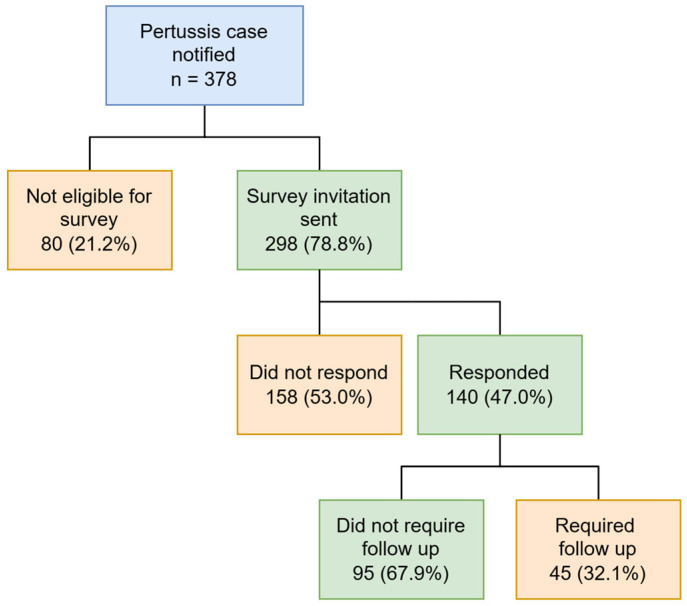
Pertussis case notifications and survey participation.

WBPHU received 378 pertussis notifications between 1 August and 31 December 2024. Of these, 202 (53.4%) were female, and the majority (63.3%) were aged between 6 and 15 years. A total of 298 cases (78.9%) met the eligibility criteria and were invited to complete the survey via SMS, with 140 responses received, giving an overall response rate of 47.0% (Figure 2). The response rate increased steadily from 10% in August to a peak of around 50% in November, before declining towards the end of December.

The median time from SMS invitation to starting the survey was 18.9 min (interquartile range: 3.1 to 111.1). The highest response rates were observed among cases aged 6–15 years and over 31 years, while no responses were received from those aged under 1 year (Table 2). There was no significant difference in response rates between males and females. The median time for survey completion was 4.9 min (interquartile range: 3.3 to 7.5).

### 3.2. Impact on Staff Workload

Among the 140 survey respondents, 95 cases (67.9%) required no further follow-up by WBPHU, while 45 (32%) needed follow-up phone calls. Significantly, the survey proved effective at identifying cases requiring urgent attention due to high-risk contacts. Before incorporation of automated prompts, 2 of the 10 follow-ups (20%) were triggered by the identification of high-risk contacts. Afterwards, this remained a primary reason for follow-up, accounting for 12 of the 35 follow-ups (34.3%), alongside antibiotic-related issues.

Case management time differed across groups. Cases not eligible for the survey took a median of 20.0 min (interquartile range: 15.0 to 25.0), while survey non-respondents had a median of 15.0 min (interquartile range: 15.0 to 20.0). Among survey respondents, those who required follow-up took a median of 20.0 min (interquartile range: 15.0 to 25.0), whereas those who required no further action took 15.0 min (interquartile range: 10.0 to 15.0). Overall, managing a survey respondent took 5 min less than a non-respondent, although this difference was not statistically significant (*p* = 0.13). This is due to some respondents still requiring phone follow-up, as a statistically significant reduction was observed between respondents who did not require follow-up and non-respondents, despite both groups having the same median time (*p* < 0.01).

### 3.3. Impact on Pertussis Incidence

Four other PHUs in Queensland shared information on their management approaches in their respective HHS catchment areas during the surge. Two continued routine phone interviews for all notified cases, while the other two limited follow-up to high-risk cases only (e.g., children under five).

**Figure 3:**
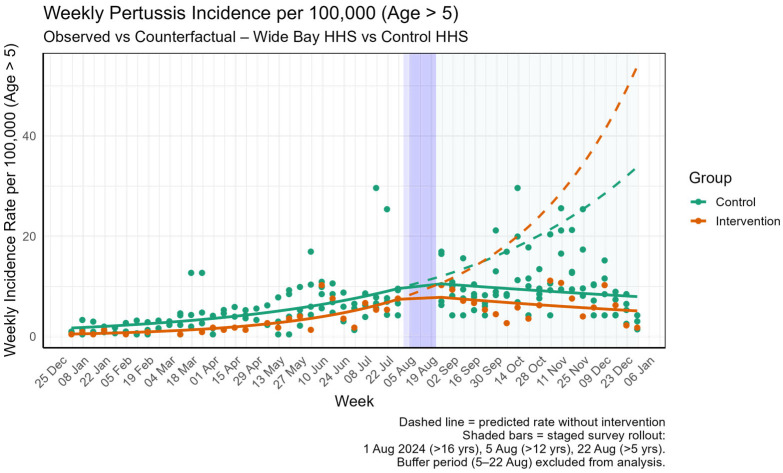
Change in level and trend of incidence of pertussis in the population aged over five years in intervention area (WBHHS) when compared to control HHSs with different management approaches.

As shown in Table 3, for the population aged over five years, the analysis revealed a statistically significant 4.0% weekly reduction in the notification trend in the WBHHS region compared to the control group (Rate Ratio: 0.96, *p* = 0.014). The immediate change in the level of notifications following the intervention was not statistically significant (Rate Ratio: 0.84, *p* = 0.383). Figure 3 visually represents this analysis, plotting the observed weekly incidence rate in WBHHS against the counterfactual projection (the predicted rate had the survey not been introduced). The plot illustrates a divergence, with the observed rate in the intervention group declining post-implementation relative to the predicted trend.

**Figure 4:**
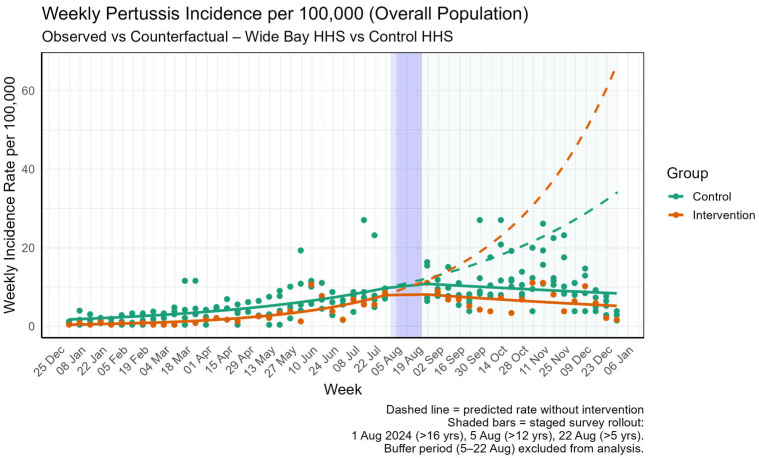
Change in level and trend of incidence of pertussis in overall population in Intervention area (WBHHS) when compared to control HHSs with different management approaches.

When the analysis was expanded to the overall population, a more pronounced and highly significant effect was observed. As detailed in Table 4, the weekly notification trend in the WBHHS region declined by an additional 4.9% per week compared to the control group (Rate Ratio: 0.95, *p* = 0.001). The immediate level change remained non-significant (Rate Ratio: 0.78, *p* = 0.197). This stronger effect is depicted in Figure 4, which shows a clear decline in the observed incidence rate for the overall population in WBHHS compared to the counterfactual trend post-intervention.

## 4. Discussion

This retrospective evaluation revealed that the integration of an online survey into the pertussis case investigation process was both well accepted and associated with several positive outcomes, including reduction in staff workload and improvement in public health response. These results are consistent with previous studies which demonstrated similar benefits of using online surveys for food-borne infections and gastroenteritis investigations [1,6,11,12,13]. By extending these findings to pertussis, this study contributes new evidence on the applicability of online surveys for the control of respiratory infections.

One key benefit observed in our evaluation was the reduction in time spent by public health staff on managing pertussis cases. Statistically significant time savings were found only among survey respondents who did not require further follow-up, suggesting that greater efficiencies could be achieved by incorporating additional automated and interactive prompts to reduce follow-up needs. Furthermore, the survey’s ability to automatically flag cases with high-risk contacts, such as pregnant women and infants under six months, demonstrates its critical value as a public health triage tool. While the absolute number of these contacts identified was small, their detection is vital for preventing severe outcomes in the most vulnerable populations. This function allows staff to bypass low-risk cases and immediately focus their expertise where it is most needed, reinforcing the survey’s role in not just saving time, but also enhancing the precision and protective impact of the public health response.

Although the per-case time savings were relatively modest, the cumulative impact could become notable during periods of high caseload. In addition, the survey could still serve as a valuable tool for cases who inadvertently required follow-up. By having the essential case information collected upfront through the survey, staff could focus their attention on managing the case-specific issues. This highlights the role of the online survey, not as a replacement but as a supportive tool that enhances the efficiency and effectiveness of the investigation process. In addition, it may help reduce staff fatigue during surge period by reducing the need for staff to conduct a large number of phone interviews.

The introduction of the online survey coincided with a noticeable flattening of the local pertussis notification curve between August and September 2024. This observation is statistically substantiated by the ITS analysis, which demonstrated a significant alteration in the disease trajectory. Specifically, for the overall population, the weekly notification trend in WBPHU declined by an additional 4.9% per week compared to control regions (*p* = 0.001). While causality cannot be definitively established from this observational data, this temporal alignment and significant trend alteration suggest that the survey may have contributed to a reduction in community transmission. Crucially, the robustness of this finding was explored through a series of sensitivity analyses (Figures S1–S4, Tables S1–S4). The significant trend change persisted even after adjusting for potential seasonality and checking for autocorrelation. Furthermore, we conducted additional analyses to address potential confounding from the control group. A formal statistical test confirmed that there was significant heterogeneity in the pre-intervention trends among the four control sites (*p* < 0.001). Given this, we compared the intervention region against each control site individually. As might be expected due to the baseline heterogeneity and reduced statistical power of these smaller comparisons, the results varied. Notably, the comparison against one control site (Control D) yielded a statistically significant weekly trend reduction of 7.1% (*p* = 0.012), consistent with our main finding. Ultimately, these additional analyses demonstrate that our primary finding is not an artefact of pooling the data and is robust, even in the presence of baseline heterogeneity, with the pooled analysis likely providing the most stable and representative estimate of the regional counterfactual trend.

By delivering immediate health information to the patient, the survey facilitated timely and appropriate implementation of antibiotic treatment and infection control measures. This advantage is particularly relevant in the context of highly transmissible respiratory infections like pertussis, where delays in communications can result in missed opportunities to prevent onward transmission.

To fully realise the survey’s potential for saving time and enhancing infection control, increasing the response rate becomes essential. While digital literacy did not appear to be a major barrier, as evidenced by a good response among older age groups, the sub-optimal response from the younger adults might have been due to concerns about scams. The absence of a government domain in the hyperlink to the Microsoft Forms survey might have further exacerbated the concern. This also highlights the importance of ensuring the credibility and security of online survey platforms as collection of personal information is involved.

Survey design also requires careful balancing between the length of the survey and the amount of information collected. Although standardised case interview forms for notifiable conditions are readily adaptable to electronic formats, incorporating dynamic and interactive elements requires additional effort and testing. Emerging technologies, such as artificial intelligence chat-bots, may help overcome some of these design and implementation challenges in the future [14].

This evaluation has several limitations. To facilitate a timely evaluation, the analysis relied on existing service data without additional data collection. Formal interviews with respondents and staff could provide further insights on survey design or implementation, such as reasons for non-response. Additionally, the time spent on cases was self-reported by PHU staff which might introduce measurement bias. Moreover, staff time data were only collected after survey deployment, which precluded any pre- versus post-survey comparison. Comparison of the nature or follow-up actions of individual pertussis cases with control PHUs was not conducted, as this would have required additional data and resources beyond the scope of the evaluation. Lastly, while the observed trend in pertussis notification rates in WBHHS was distinct from other HHSs, other confounding factors may have contributed to these differences. Furthermore, the ITS analysis was based on a seven-month pre-intervention period within a single year. The lack of a longer historical time series to establish a more stable baseline trend may have resulted in a more extreme counterfactual projection. Future analyses incorporating multi-year data would be beneficial to confirm these findings and better control for long-term cyclical patterns. Finally, the under-five age group was not included in the ITS analysis, as the number of cases in this stratum was too low to support a statistically stable time series analysis.

Following the evaluation, WBPHU made several enhancements to the survey. In early 2025, the survey was migrated to Queensland Health’s REDCap platform [15,16], which allowed for more interactive components which could reduce need for follow-up and further improve time-saving. This platform also supported the automated and formatted reporting of responses to PHU staff and the use of a government domain in the survey hyperlink, which might address some of the previous concerns. The online survey approach was also being expanded to support case investigations for other notifiable conditions, such as food-borne infections, with the aim of improving the efficiency and timeliness of public health responses.

## 5. Conclusions

This evaluation demonstrates that an online survey can be a practical, well-accepted, and effective supplement to current case investigation processes for pertussis in a regional public health setting. The survey was associated with tangible reductions in staff workload for a majority of respondents and coincided with a favourable and statistically significant alteration in community disease transmission trends. While challenges in optimising response rates and survey design remain, the online survey approach offers significant potential to enhance the efficiency and timeliness of managing pertussis and other notifiable conditions, particularly during outbreaks or periods of high demand on public health services.

## Figures and Tables

**Table 1:** Study objectives, data sources, and metrics.

Objectives	Data Sources	Metrics
1. Evaluate the online survey responses made by pertussis cases or their parents	NoCS Online survey response data	Survey response rate by age, sex, and date of notification Time to survey response and completion
2. Assess impact of the online survey on staff workload	WBPHU case follow-up records	Proportion of cases requiring follow-up Time spent managing each case, stratified by different case categories
3. Determine whether the survey implementation improved pertussis control in Wide Bay region	NoCS Survey with other PHUs Australian Bureau of Statistics	Trends of pertussis notification rates (1 January 2024 and 31 December 2024) in WBPHU compared with groups of other PHUs that conduct pertussis case investigation without using the online survey in their respective HHS catchment areas

**Table 2:** Response rate by sex and age groups.

Characteristics	Response Rate (%)
Sex	
Female	48.4
Male	44.4
Age group	
<1	0.0
1–5	25.0
6–10	45.8
11–15	48.5
16–20	19.0
21–30	30.0
31–40	61.9
41–50	47.4
51–60	66.7
61–70	63.6
71–80	77.8

**Table 3:** Intervention effect on pertussis incidence in the population aged over five years. Level and Trend Change (Intervention vs. Control, Age > 5).

Effect	Log Coefficient	95% CI	Rate Ratio	% Change	*p*-Value
Level change (Intervention vs. Control)	−0.173	(−0.561, 0.216)	0.84	−15.9%	0.383
Trend change (Intervention vs. Control)	−0.041	(−0.074, −0.009)	0.96	−4%	0.014

**Table 4:** Impact on weekly incidence rate of pertussis in overall population. Level and trend change (Intervention vs. Control, overall population).

Effect	Log Coefficient	95% CI	Rate Ratio	% Change	*p*-Value
Level change (Intervention vs. Control)	−0.242	(−0.61, 0.127)	0.78	−21.5%	0.197
Trend change (Intervention vs. Control)	−0.050	(−0.081, −0.02)	0.95	−4.9%	0.001

## Data Availability

The data presented in this study are available on request from the corresponding author due to privacy and ethical restrictions.

## Supplementary Materials

The following supporting information can be downloaded at: https://www.mdpi.com/article/10.3390/tropicalmed10090260/s1, Figure S1: Autocorrelation and Overdispersion of Model Results: Age > 5 Years; Figure S2: Seasonally Adjusted ITS Plot (Age > 5); Figure S3: Autocorrelation and Overdispersion of Model Results: Overall Population (All Ages); Figure S4: Seasonally Adjusted ITS Plot (All Ages); Figure S5: Flowchart of the workflow and components of the online survey; Table S1: Seasonally Adjusted ITS Estimates (Age > 5); Table S2: Seasonally Adjusted ITS Estimates (All Ages); Table S3: Statistical Test for Homogeneity of Pre-Intervention Trends (>5 Years Population); Table S4: Statistical Test for Homogeneity of Pre-Intervention Trends (Overall Population).

## Abbreviations

The following abbreviations are used in this manuscript:

HHS	Hospital and Health Service
ITS	Interrupted Time Series
NoCS	Notifiable Conditions System
PHU	Public Health Unit
SMS	Short Message Service
WBHHS	Wide Bay Hospital and Health Service
WBPHU	Wide Bay Public Health Unit
