# Supplementary material for: Evaluation of an Online Survey for Pertussis Case Investigations in Regional Queensland: Impacts on Workload and Disease Trends
Source: Trop Med Infect Dis. 2025 Sep 12;10(9):260. doi: 10.3390/tropicalmed10090260 (PMC12474398; doi:10.3390/tropicalmed10090260)
Supplement: Supplementary file 1 [file tropicalmed-10-00260-s001.zip › tropicalmed-3839469-supplementary.pdf]

Supplementary results for ITS analysis

Supplementary Figure 1: Autocorrelation and Overdispersion of model Results: Age >5 Years

Overdispersion ratio (Age >5): 4.13

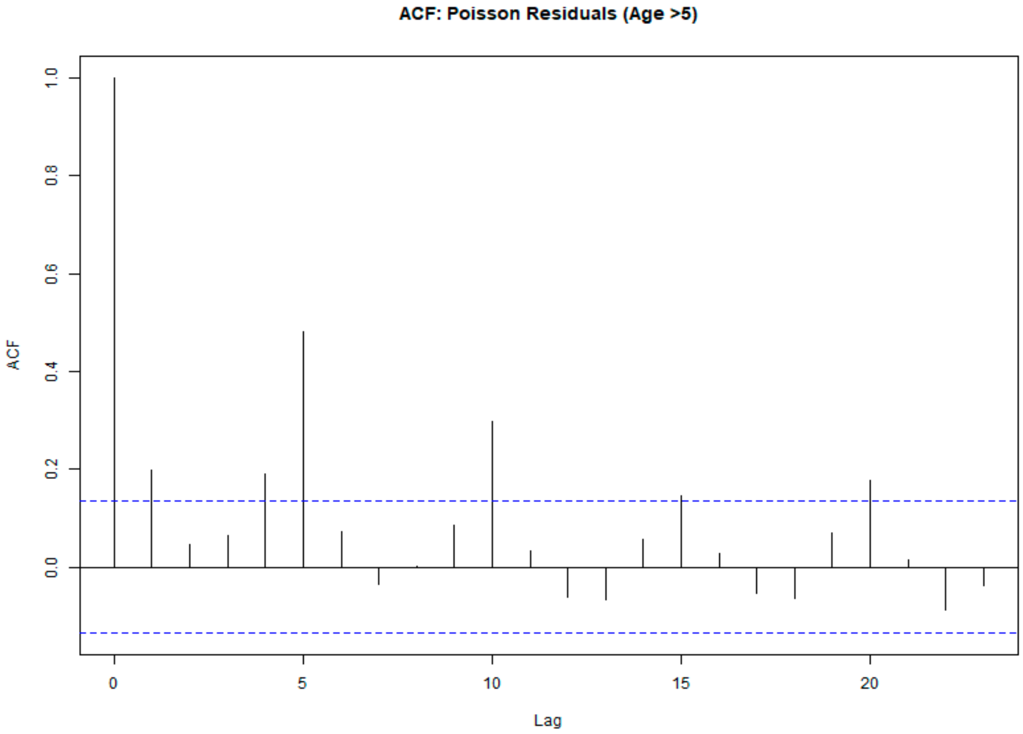

Supplementary Figure 2: Seasonally Adjusted ITS Plot (Age >5)

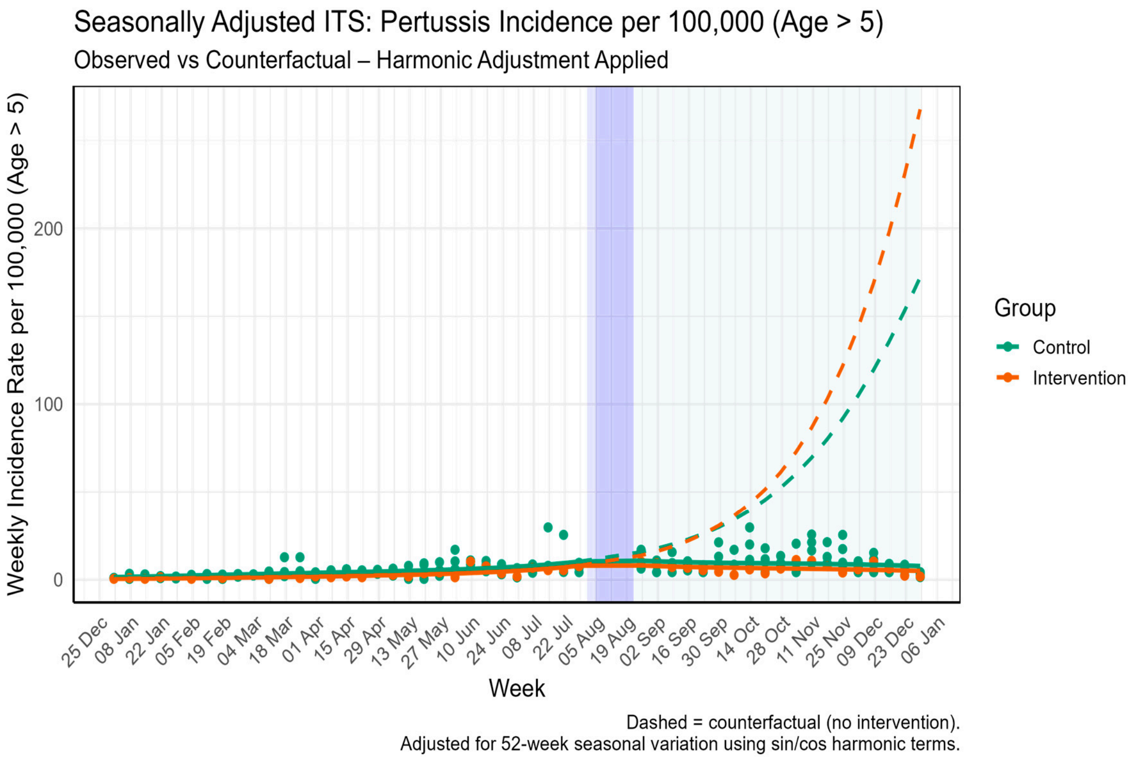

Supplementary Figure 3: Autocorrelation and Overdispersion of model Results: Overall Population (All Ages)

Overdispersion ratio (All Ages): 4.45

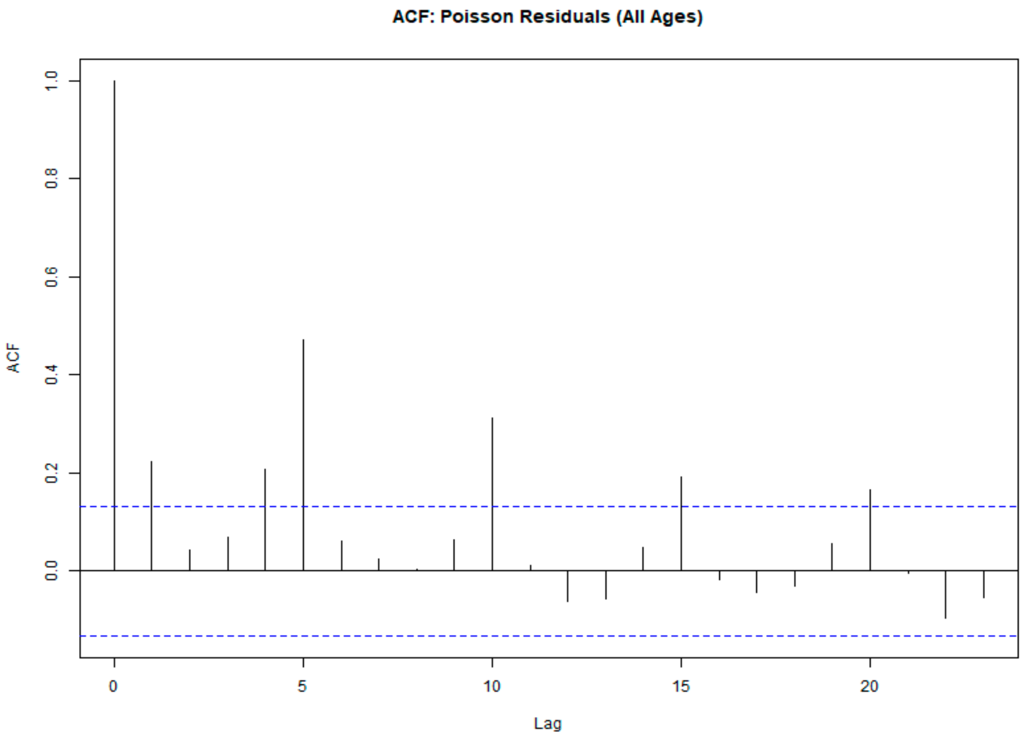

Supplementary Figure 4: Seasonally Adjusted ITS Plot (All Ages)

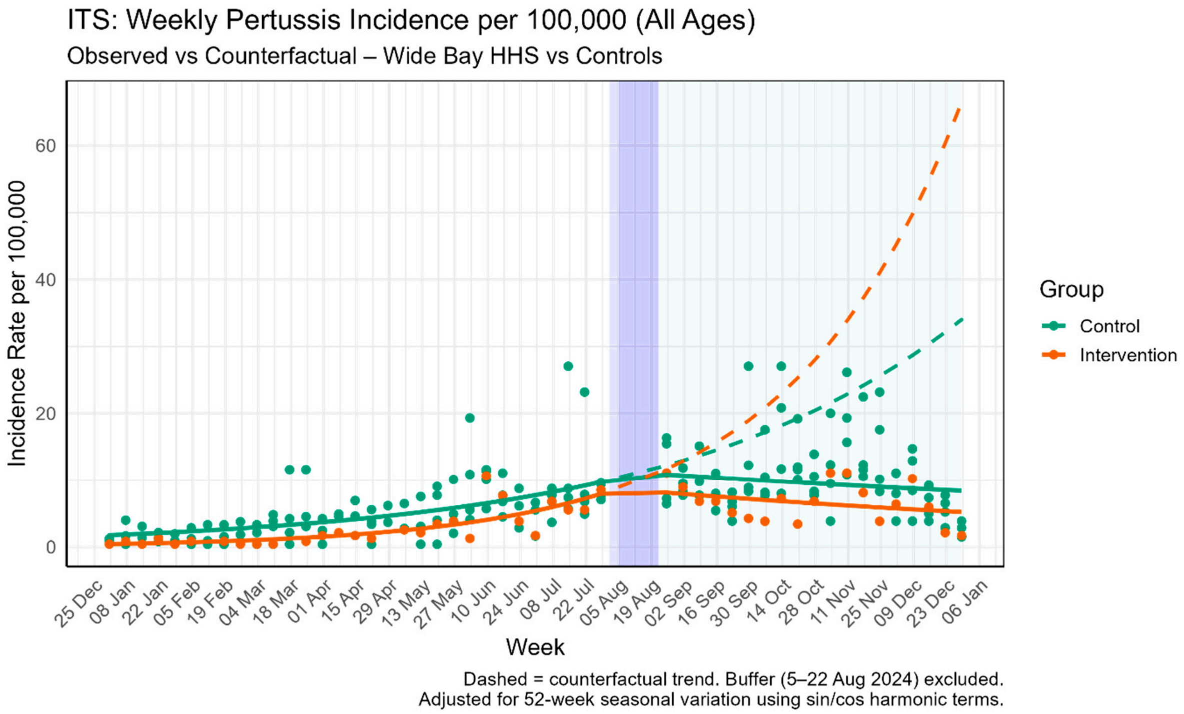

Supplementary Figure 5: Flowchart of the workflow and components of the online survey.

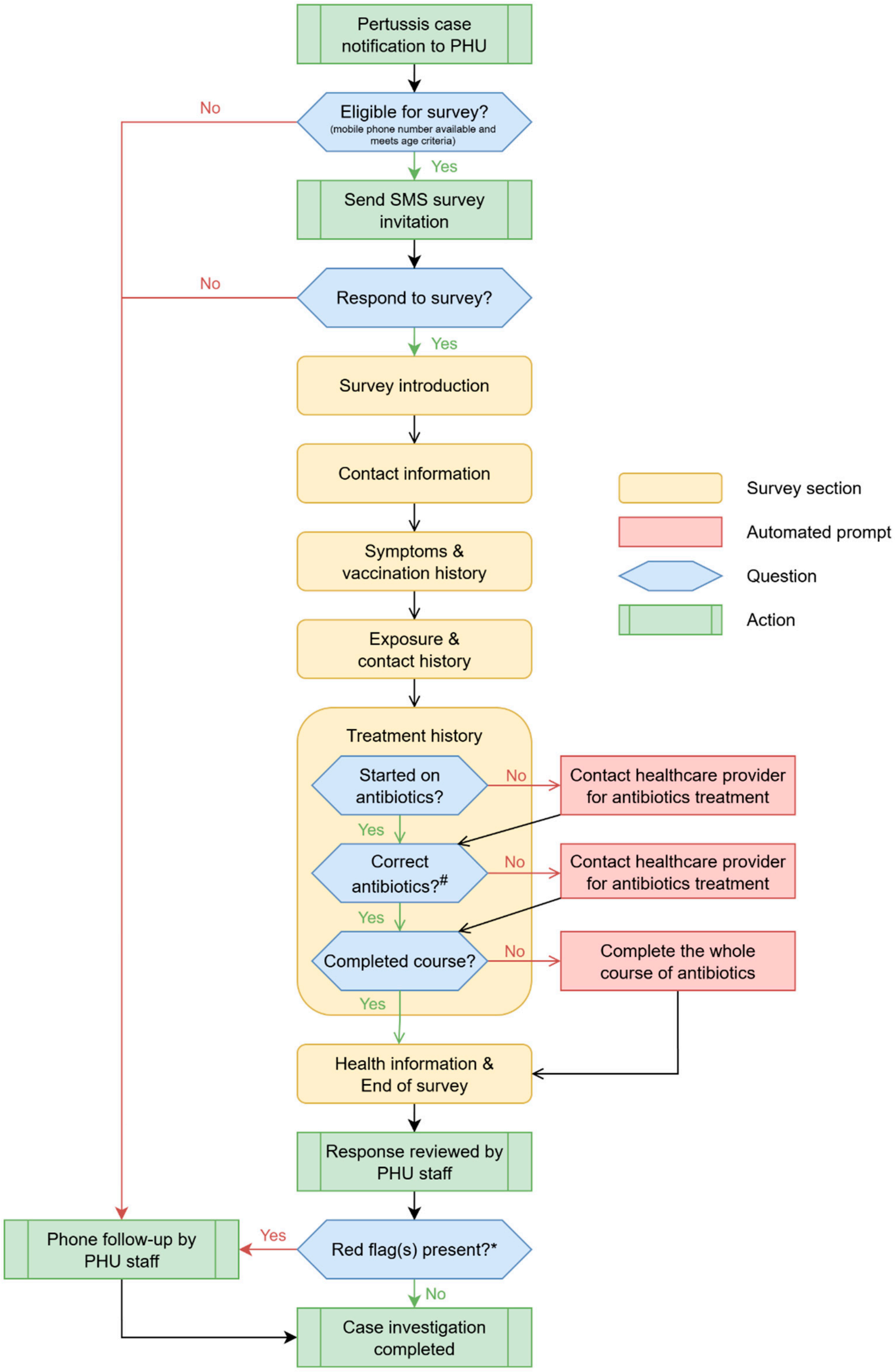

As recommended by the Australia CDNA National Guidelines for Public Health Units

* High-risk exposures during the infectious period, including household contacts with women in the last month of pregnancy or children under six months of age, or attendance at high-risk settings such as childcare centres, preschools, schools, residential care facili-ties, or healthcare facilities (excluding attendance for the current pertussis illness)

Supplementary Table 1: Seasonally Adjusted ITS Estimates (Age >5)

Effect	Log Coefficient	95% CI	Rate Ratio	% Change	P-value
Level change (seasonal)	-0.165	(-0.554, 0.224)	0.85	-15.2%	0.404
Trend change (seasonal)	-0.040	(-0.073, -0.008)	0.96	-3.9%	0.016

Supplementary Table 2: Seasonally Adjusted ITS Estimates (All Ages)

Effect	Log Coefficient	95% CI	Rate Ratio	% Change	P-value
Level change (seasonal)	-0.227	(-0.593, 0.141)	0.80	-20.3%	0.225
Trend change (seasonal)	-0.049	(-0.08, -0.019)	0.95	-4.8%	0.002

Supplementary Table 3: Statistical Test for Homogeneity of Pre-Intervention Trends (>5 Years Population)

Comparison	Log Coefficient	Std. Error	P-value
Trend of Control B vs. Control A	-0.101	0.026	<0.001
Trend of Control C vs. Control A	-0.044	0.045	0.327
Trend of Control D vs. Control A	-0.096	0.027	<0.001

An omnibus Likelihood Ratio Test for the overall homogeneity of trends was statistically significant (p = <0.001).

Supplementary Table 4: Statistical Test for Homogeneity of Pre-Intervention Trends (Overall Population)

Comparison	Log Coefficient	Std. Error	P-value
Trend of Control B vs. Control A	-0.094	0.023	<0.001
Trend of Control C vs. Control A	-0.031	0.042	0.459
Trend of Control D vs. Control A	-0.092	0.024	<0.001

An omnibus Likelihood Ratio Test for the overall homogeneity of trends was statistically significant (p = <0.001).
